# Veratria in Facial Neuralgia

**Published:** 1844-04

**Authors:** 


*Veratria in Facial Neuralgia.*—An ointment composed of veratria, one part, to eighty parts of lard, has been found very useful as an external application in cases of facial neuralgia. But the preparation is much more efficacious if made with rancid instead of fresh lard, which is probably owing to a salification and greater solubility effected in the veratria by the agency of the free acid in the fat.—*Braithwaite's Retrospect.*

